# Diagnostic value of ultrasonography for the detection of disc displacements in the temporomandibular joint: a systematic review and meta-analysis

**DOI:** 10.1007/s00784-018-2359-4

**Published:** 2018-02-17

**Authors:** Naichuan Su, Arjen J. van Wijk, Corine M. Visscher, Frank Lobbezoo, Geert J. M. G. van der Heijden

**Affiliations:** 1 State Key Laboratory of Oral Disease, West China Hospital of Stomatology, Sichuan University, Chengdu, China; 2 Department of Prosthodontics, West China Hospital of Stomatology, Sichuan University, Chengdu, China; 3 Department of Social Dentistry, Academic Centre for Dentistry Amsterdam, University of Amsterdam and Vrije Universiteit, 1081 LA, Amsterdam, The Netherlands; 4 Department of Oral Kinesiology, Academic Centre for Dentistry Amsterdam, MOVE research Institute Amsterdam, University of Amsterdam and Vrije Universiteit, Amsterdam, The Netherlands

**Keywords:** Temporomandibular joint disorders, Ultrasonography, Meta-analysis, Magnetic resonance imaging

## Abstract

**Objectives:**

The aim was to assess the added diagnostic value of ultrasonography (US) for establishing the presence or absence of disc displacements (DDs) in temporomandibular joints (TMJs).

**Materials and methods:**

Pubmed and EMBASE were searched electronically to identify diagnostic accuracy studies that assessed the diagnostic value of US for the diagnosis of DD, using Magnetic resonance imaging (MRI) as the reference standard. Meta-analyses were performed with Metadisc 1.4 and RevMan 5.3.

**Results:**

A total of 16 studies qualified for meta-analyses. For the diagnosis of DD at closed mouth position (DD-CM) and DD at maximum mouth-opening position (DD-MMO), the added values of a positive result with US for ruling in DD-CM and DD-MMO were 22 and 41%, while those of a negative result with US for ruling out DD-CM and DD-MMO were 30 and 20%. For the diagnosis of DD with reduction (DDWR) and DD without reduction (DDWoR), the added values of a positive result in US for ruling in DDWR and DDWoR were 35 and 41%, while those of a negative result in US for ruling out DDWR and DDWoR were 21 and 27%.

**Conclusions:**

Using MRI as reference standard, the added values of both positive predictive values and negative predictive values of US for ruling in and ruling out DDs are sufficient in the decision-making in dental practice.

**Clinical relevance:**

US can be a good imaging tool to supplement clinical examination findings in patients with suspected DDs. Combined static and dynamic examinations using high-resolution US should be preferred.

## Introduction

Temporomandibular disorders (TMDs) are a type of musculoskeletal disorders with pain that compromise masticatory muscles, temporomandibular joints (TMJs), and other anatomical structures in the orofacial region [1]. Disc displacement (DD), i.e., an abnormal location of the TMJ disc, is a highly prevalent derangement within the TMJs [2]. The prevalence of DD in general population ranges from 18 to 35% [2], while that in TMD patients ranges from 38 to 73% [3–5]. DD is categorized based on the relation of the displaced disc with the mandibular condyle. The displacement of the disc can be anterior, anterolateral, anteromedial, lateral, medial, and posterior [6]. DD can be classified as DD with reduction (DDWR) or DD without reduction (DDWoR) [7]. DDWR was thought to be the most common internal derangement within the human TMJs [8]. In the general population, the prevalence of DDWR is about 40%, while that of DDWoR is 4% [9]. In TMD patients, the prevalence of DDWR ranges from 24 to 47%, while that of DDWoR ranges from 11 to 26% [3–5]. In DDWR, which is the most frequent type, the displaced disc returns to the normal position on mouth opening, thereby producing a “reciprocal click,” while DDWoR is a non-clicking condition that may be accompanied by a limited mouth opening and a deviation of the jaw to the affected side [7]. It is reported that DDWR is mostly a stable, pain-free, and lifelong condition of the joint [2]. However, in a small minority of patients, the disc loses its capacity to reduce on opening [2]. The loss of disc reduction may be accompanied by signs and symptoms of a closed lock-like painful and limited mouth opening [2]. A disc displacement may be a progressive disorder of the TMJ, starting as a DDWR early upon opening, then progressing to a disc displacement with a reduction late upon opening and finally, to a DDWoR [2]. However, there are indications that DDWR or DDWoR may either retard or arrest condylar growth and may thus be related to mandibular retrognathia and/or facial asymmetry, though these skeletal consequences are probably rare and small [2].

Following clinical examination, imaging of the TMJ can be used to confirm a possible disc displacement when indicated [10]. A proper diagnosis may inform the decision on its management [11]. In such instances, magnetic resonance imaging (MRI) is one of the most common imaging tools. It is in general widely regarded as the most accurate method, because it displays the anatomic details of the TMJ and high sensitivity and specificity of MRI have been reported [12, 13]. However, it has several drawbacks in clinical practice. MRI cannot be carried out in patients with pacemakers and metallic prosthesis or claustrophobic patients [14, 15]. Also, the use of MRI is limited by the required centralized facilities, the high cost, and the long time it takes for scanning compared to other imaging modalities like computed tomography (CT) [15]. Recently, visualization of the TMJ and its disc with US has gained attention and importance for by both research and patient care, because it is less expensive, less time-consuming, and easily accessible [16]. Also, it can be used to directly observe the joint disc movement during opening and closing of the mouth, which is considered an advantage, because it allows the investigator to detect disc position more accurately [17].

Although US is not yet commonly used in the diagnosis of TMJ DD, it has gained considerable attention over the last few years. However, its use in clinical practice is complicated by the wide and unexplained variation of the reported sensitivity, specificity, and accuracy, ranging from 13 up to 100%, from 62 up to 100%, and from 52 up to 100%, respectively. Moreover, the (added) diagnostic value of US compared to that of patients before receiving US for ruling in and ruling out the DD in clinical practice has not been established.

Thus, the purpose of this systematic review of clinical diagnostic outcome studies is to establish the diagnostic accuracy of US in patients with suspected of TMJ DD. The findings of this review will provide a summary on the utility of US in ruling in and ruling out DD. As such, the added diagnostic value that will be reported may facilitate decision-making on patient in daily practice.

## Materials and methods

This systematic review and meta-analyses are carried out based on the Preferred Reporting Items for Systematic Reviews and Meta-Analyses: The PRISMA Statement [18].

### Search strategy

Relevant publications were searched in electronic bibliographic sources, including PUBMED and EMBASE, without language restriction, up to May 2016. Relevant dental journals and reference lists of included studies were manually searched to avoid selection bias.

A combination of free text words and systematic vocabulary (Medical Subject Headings, EMTREE terms and topics) was used in the search strategies. The search strategy is presented in Table 1.

**Table 1:** The search strategies for the included studies

Database	Search strategies
Pubmed	(“Ultrasonography” [Mesh] OR “ultrasonography” [All fields] OR “ultrasound” [All fields] OR “sonography” [All fields]) AND (“Temporomandibular joint disorders” [Mesh] OR “Temporomandibular joint disorders” [All Fields] OR “Temporomandibular joint disc” [Mesh] OR “Temporomandibular joint disc” [All Fields] OR “Temporomandibular joint dysfunction syndrome” [Mesh] OR “Temporomandibular joint dysfunction syndrome” [All Fields] OR “Temporomandibular joint” [Mesh] OR “Temporomandibular joint” [All Fields] OR “Craniomandibular disorders” [Mesh] OR “craniomandibular disorders” [All fields])
EMBASE	(“echography” or ultrasonography or ultrasound or sonography) and (“temporomandibular joint” or “jaw disease” or “temporomandibular joint disorder” or (temporomandibular and joint and disc) or (temporomandibular and joint and dysfunction and syndrome) or (craniomandibular and disorders))

### Selection criteria

Only publications reporting studies satisfying the following criteria were included:

1. Evaluating the diagnostic accuracy of US for DDs;
2. Patients with any symptoms or clinical signs related to a DD without considering type of displacement, gender, age, race, and social economic status;
3. MRI used as the reference test;
4. False-positive (FP), true-positive (TP), false-negative (FN), and true-negative (TN) values of US based on MRI for the diagnosis of DD are reported or sufficient reporting of data to (re)calculate these.

For US, a normal disc position has been defined for the closed-mouth position as the location of the intermediate zone of the disc between the anterosuperior aspect of the mandibular condyle and the posteroinferior aspect of the articular eminence, while for the open-mouth position, it is the location of the intermediate zone of the disc between the condyle and the articular eminence. DD is seen when compared to the above, the intermediate zone of the disc is positioned to the anterior, anteromedial, anterolateral, medial, or lateral [17, 19].

On MRI, a normal disc position is seen when the posterior band of the disc is located between 12 and 3 o’clock or in superior position relative to the condyle. DD is defined when the posterior band of the disc is in an anterior, anteromedial, anterolateral, medial, or lateral position relative to the superior part of the condyle [17].

DDWR is defined as displaced position of the disc in relation to the superior part of the condyle in the closed-mouth position and normal disc position in the open-mouth position. DDWoR is defined as displaced position of the disc in relation to the superior part of the condyle in both closed- and open-mouth positions.

Based on US, the disc can be identified as hyperechoic, hypoechoic, and isoechoic. Hyperechoic is defined that the disc is highly reflective and echo rich when compared with neighboring structures and appears as varying shades of lighter gray. Hypoechoic is defined that the disc is less reflective and low amount of echoes when compared with neighboring structures and appears as varying shades of darker gray. Isoechoic is defined that the disc has similar echogenicity to a neighboring structure [20].

Studies which met the following criteria were excluded:

1. Case-control studies, review articles, and case reports;
2. Duplicate publications.

Two reviewers (SN and LY) independently assessed the titles and abstracts of all identified studies from the electronic searches. Full texts were obtained for studies that met the inclusion criteria or where a clear decision could not be made from the title and abstract alone. In the latter case, selection was based on full text reading. During subsequent quality assessments and data extraction, the reviewers were neither masked for the study findings and reported conclusions, nor for the identity of the journal, the authors, and their affiliations. Reviewers resolved initial disagreements by consensus discussion.

### Data extraction

For all included studies, the following data were extracted using a standardized form: (a) authors` names; (b) centers involved in the study and their locations; (c) year of publication; (d) demographic characteristics of participants, such as age and gender; (e) study design features; (f) inclusion and exclusion criteria; (g) diagnostic criteria; (h) type of US: US was performed at closed-mouth position and maximum-mouth-opening position (static) and/or US was performed during the mouth opening movement (dynamic); (i) the US positioning (horizontal or vertical); and (j) US resolution (high or low). High-resolution US (HRUS) is defined as the resolution of US being 12 MHz or more, while low-resolution US (LRUS) is defined as the resolution being lower than 12 MHz [15].

In addition, we aimed to extract the total number of study patients included and analyzed, as well as those with a true-positive, false-positive, false-negative, and true-negative finding of US based on MRI for the diagnosis of DD. These were either reported or recalculated from the reported data and explored missing data. Thereafter, we recalculated the prevalence of DD according to MRI and US, as well as the positive predictive value (PPV), negative predictive value (NPV), sensitivity, and specificity. PPV was defined as the risk of the presence of DD based on MRI in those with presence of DD based on US (TP/(TP + FP)). NPV was defined as the risk of the absence of DD based on MRI in those with absence of DD based on US (TN/(FN + TN)). Sensitivity was defined as the percentage of patients with a DD, as based on MRI, correctly identified as such based on US (TP/(TP + FN)), while specificity was defined as the percentage of patients free of a DD, as based on MRI, correctly identified as such based on US (TN/(FP + TN)). The added value of US for ruling in the DD is calculated by subtracting the prior probability (prevalence) from the PPV. The added value of US for ruling out the DD is calculated by subtracting the complement of the prevalence (1 minus the prior probability) from the NPV.

### Quality assessment

The QUADAS-2 tool was used to assess the methodological quality of the diagnostic accuracy studies [21]. The tool includes four domains, including patient selection, index test, reference standard, and flow and timing. Each domain was assessed in terms of risk of bias, with the first three domains also considered in terms of applicability concerns. A “Risk of bias” judgment (“high,” “low,” or “unclear”) was made for each domain. If the answers to all signaling questions within a domain were judged as “yes” (indicating low risk of bias for each question), the domain was judged to be at low risk of bias. If any signaling question was judged as “no” (indicating a high risk of bias), the domain was scored as a high risk of bias. This was followed by a judgment about concerns regarding clinical applicability for the patient selection, index test, and reference standard domains. Concerns about applicability were rated as “low,” “high,” or “unclear.” High concern was rated when the study did not match the review question in any domain, while low concern was rated when the study matched the review question in each domain. Unclear domain was rated only when insufficient data were reported.

Two independent raters, blinded for each other’s ratings, performed the QUADAS-2 assessment. Reviewers resolved initial disagreements by consensus discussion.

### Statistical analysis

The data analysis was conducted using RevMan software, version 5.3 (RevMan, Copenhagen, Denmark; the Nordic Cochrane Centre, the Cochrane Collaboration, 2008), and Meta-Disc 1.4 (Meta-Disc, Clinical Biostatistics Unit, Ramon y Cajal Hospital, Madrid, Spain).

A random-effect model was used to calculate the overall pooled estimates of sensitivity, specificity, positive likelihood ratio (LR+), negative likelihood ratio (LR−), and diagnostic odds ratio (DOR) with 95% CIs. The forest plot and summary receiver operating characteristic (SROC) curve were generated to graphically present the interaction between sensitivity and specificity. The overall test performance was quantified using the area under the SROC curve (sAUC) and Q* (the point where sensitivity equals specificity on the SROC curve). sAUC measures the overall capacity of the test to discriminate between participants with the disease and those without it. A sAUC of 0.5 or lower indicates no discriminative ability. The higher a sAUC is, the higher the diagnostic accuracy of US is.

Finally, clinical value of US was assessed using the prevalences (prior probabilities) and the posterior probabilities, being the positive predictive values (PPV) and negative predictive values (NPV). The PPVs and prevalences were used to assess the (added) diagnostic value of US for ruling in an increased risk of DD. The NPVs and complement of the prevalences (1 minus prevalence) were used to assess the (added) diagnostic value of US for ruling out an increased risk of DD.

## Results

### Results of search and selection

**Fig. 1:**
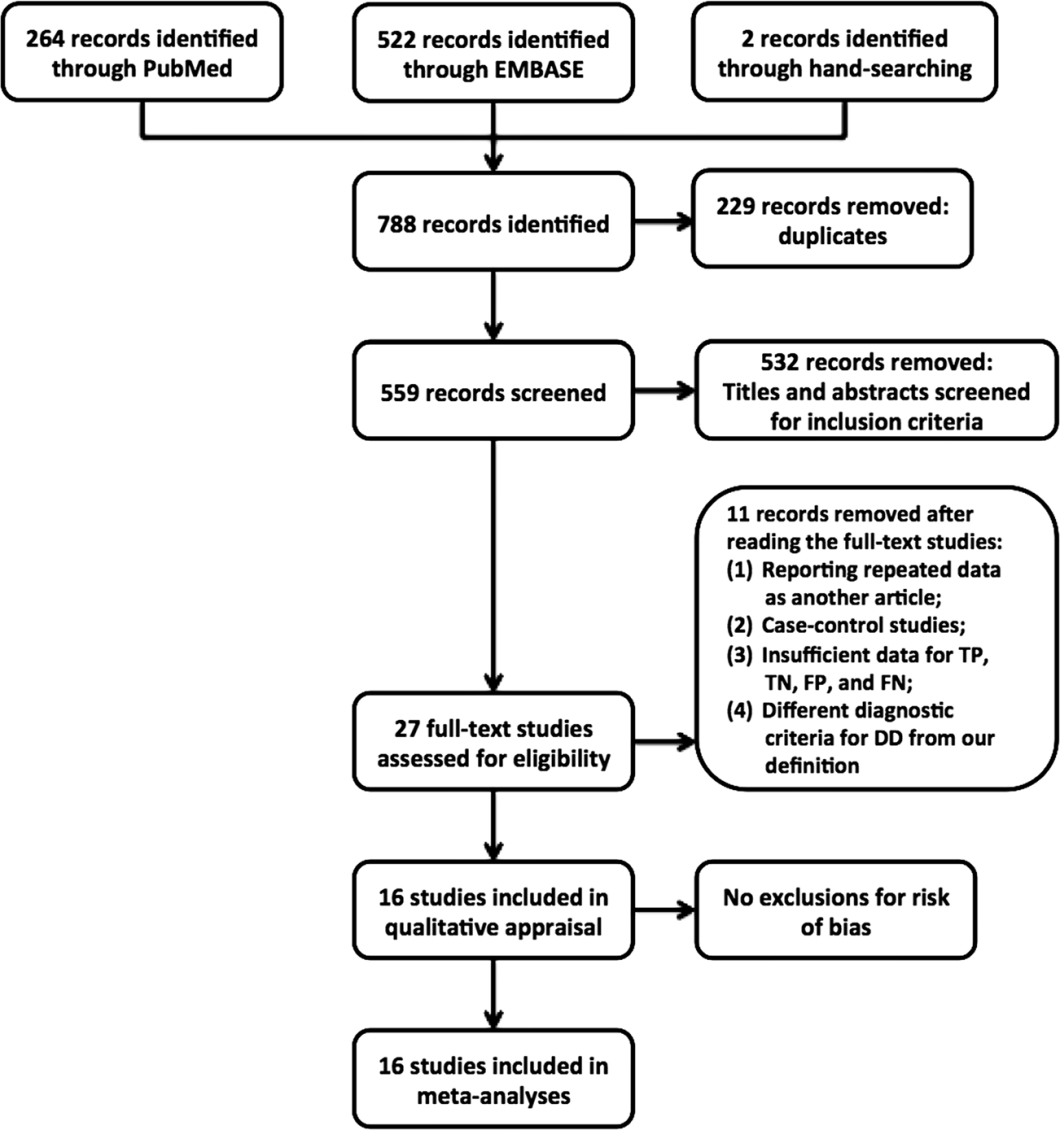
Flow diagram of study inclusion

The initial search identified a total of 559 studies. During screening the titles and abstracts, 532 of them did not satisfy the inclusion criteria. Another 11 studies eventually did not satisfy the inclusion criteria after carefully reading the full text publications. Therefore, a total of 16 studies were included in the present review (Fig. 1) [17, 19, 22–35].

### Characteristics of included studies

With respect to the design of the included studies, all of the 16 studies were prospective follow-up studies. The centers involved in the diagnostic assessments were various. Seven of the included studies were performed in the University of Innsbruck, Austria [19, 22, 24, 26, 28, 31, 33], and another two were performed in Frankfurt University Medical Center, Germany [23, 30]. The interpretation of US imaging varied among these studies. Eight studies identified the disc as hypoechoic to isoechoic [23, 24, 26, 27, 29–31, 33] and another three studies as hyperechoic [17, 25, 32]. However, the remaining five studies did not report the radiographic features of disc in US. The other characteristics are presented in Table 2.

**Table 2:** Characteristics of included studies

Ultrasonography (US)															
Study ID	Country	Center and date	Patient characteristics	Number of TMJs	Inclusion criteria	Transducer (MHz)	Type of US	Initial Positioning	Radiographic features of disc in US	MRI scanner (T)	Outcome	TP	TN	FP	**FN**
Jank 2005 [22]	Austria	University of Innsbruck/date is not mentioned	100 patients; aged ≥ 16 years	200	Patients with indefinite diagnosis of TMD, failed therapy, or need for presurgical MRI	Unclear	Static and dynamic	Unclear	Unclear	2.2 T	DD-CM	127	57	5	11
											DD-MMO	66	113	11	10
Brandlmaier 2003 [19]	Austria	University of Innsbruck/date is not mentioned	48 patients (35 Females/13 Males); 17–67 years, mean 38 years	96	Patients with reported pain and dysfunction of TMJ region	12 MHz	Static	60 degrees to the Frankfort plane	Unclear	1.5 T	DD-CM	53	30	4	9
											DD-MMO	20	57	12	7
											DDWR	19	55	6	16
											DDWoR	20	57	12	7
Bas 2011 [17]	Turkey	Ondokuz Mayis University/date is not mentioned	91 patients (74 Females/17 Males); mean 25 years	182	Patients with clinical diagnosis of ID	10 MHz	Static and dynamic	Horizontal and vertical	A thin area of hyperechogenicity surrounded by a hypoechoic halo	0.5 T	DD-CM	95	34	12	41
Landes 2006 [23]	Germany	Frankfurt University Medical Centre/July 2002 to May 2003	68 patients (44 Females/24 Males); 14–77 years, mean 32 years	136	Patients with reported pain and dysfunction of TMJ areas	8–12.5 MHz	Static, 3D	Horizontal	A hypoechogenic band overlying the lateral condylar pole	1.5 T	DD-CM	29	36	22	18
											DD-MMO	9	73	13	12
Jank 2001 [24]	Austria	University of Innsbruck/date is not mentioned	66 patients (51 Females/13 Males); 13–78 years, mean 38.2 years	132	Patients with a clinical diagnosis of ID	12 MHz	Static	Horizontal and vertical	A hypoechogenic structure surrounded by a hyperechogenic rim	1.5 T	DD-CM	68	35	10	19
											DD-MMO	33	69	9	21
Habashi 2015 [25]	Israel	Bruce and Ruth Rappaport School of Medicine/March 2011 to October 2011	39 patients (26 Females/13 Males); 18–77 years, mean 37.23 ± 16.26 years	78	Patients with symptoms and signs of TMJ disorders	5–17 MHz	Static and dynamic	Horizontal and vertical	A central hyperechoic lines surrounded by a shadow hypoechoic rim	3 T	DD-CM	19	36	16	7
											DD-MMO	11	53	7	7
											DDWR	33	24	12	9
											DDWoR	24	33	9	12
Emshoff 2002 (a) [26]	Austria	University of Innsbruck/December 1997 to September 2000	208 patients (159 Females/49 Males); 13–78 years; mean 38.5 years	341	Patients with symptoms of TMJ ID	12 MHz	Static	Vertical	A thin isoechogenic band lying adjacent to the condyle	1.5 T	DD-CM	176	104	16	45
											DD-MMO	97	184	15	45
Yang 2012 [27]	China	The First Hospital of Neijiang/June 2008 to June 2011	35 patients (20 Females/15 Males); 19–63 years, mean 37.3 ± 9.6 years	40	Patients with pain or dysfunction in the TMJ regions	12 MHz	Static	Vertical	A thin hypoechogenic band	1.5 T	DD-CM	24	9	1	6
											DD-MMO	22	16	1	1
											DDWoR	19	16	1	4
Emshoff 1997 [28]	Austria	University of Innsbruck/date is not mentioned	17 patients (14 Females/3 Males); 16–60 years, mean 33.8 years	34	Patients with signs and symptoms of TMD	7.5 MHz	Static and dynamic	Horizontal	Unclear	1.5 T	Static DD-CM	13	5	2	13
											Static DD-MMO	1	14	5	7
											Dynamic DD-CM	10	7	0	16
											Dynamic DD-MMO	1	18	1	7
Razek 2015 [29]	Egypt	Mansoura Faculty of Dentistry/date is not mentioned	20 patients (17 Females/3 Males); 15–57 years, mean 26.1 years	40	Patients with symptoms of TMJ ID	12 MHz	Static	Horizontal and vertical	A hypoechogenic band	1.5 T	DD-CM	23	8	3	7
Landes 2007 [30]	Germany	Frankfurt University Medical Centre/May 2004 to July 2004	33 patients (23 Females/10 Males); 14–77 years, mean 34 years	66	Patients with reported pain and dysfunction of TMJ areas	8–12.5 MHz	Static, 2D, 3D	Horizontal	A hypoechogenic band	1.5 T	2D DD-CM	19	17	19	11
											2D DD-MMO	8	40	11	7
											3D DD-CM	20	19	17	10
											3D DD-MMO	8	42	9	7
Emshoff 2002(b) [31]	Austria	University of Innsbruck/June 2000 to March 2001	64 patients (55 Females/9 Males); 17–65 years, mean 35.5 years	128	Patients with TMJ ID	12 MHz	Dynamic	Vertical	A thin hypo-to-isoechoic band lying adjacent to the inferior relation	1.5 T	DDWR	22	96	5	5
											DDWoR	50	65	3	10
Tognini 2005 [32]	Italy	University of Pisa/date is not mentioned	41 patients	82	Patients with at least one of the clinical signs including joint sounds, joint pain or restricted or deviated mandibular function	8–20 MHz	Static and dynamic	Vertical	A thin area of hyperechogenity surrounded by a hypoechoic halo	1.5 T	DD-CM	17	33	8	10
Emshoff 2003 [33]	Austria	University of Innsbruck/date is not mentioned	48 patients (40 Females/8 Males); 15–72 years, mean 51 years	96	Patients with clinical diagnosis of TMD	12 MHz	Dynamic	Vertical	A thin hypoechoic-to-isoechoic band lying adjacent to the inferior relations of the mandibular condyle	1.5 T	DDWoR	41	48	5	2
Kaya 2010 [34]	Turkey	Ankara Physical Medicine and Rehabilitation Education and Research Hospital/ date is not mentioned	52 patients (47 Females/5 Males); mean 28.30 ± 10.76 years	52	Patients with unilateral chronic TMJ pain	7.5 MHz	Static and dynamic	Horizontal and vertical	Unclear	1.5 T	DD-CM	42	1	5	4
											DDWR	22	8	13	9
											DDWoR	8	32	4	8
Starke 2011 [35]	Germany	Faculty of Economic and Social Sciences, Osnabruck/September 2010 to November 2010	22 patients (16 Females/6 Males); 20–70 years, mean 48 years	44	Patients with a diagnosis of craniomandibular dysfunctions	4–13 MHz	Static and dynamic	Vertical	Unclear	1.5 T	DD-CM	17	12	7	0

### Quality assessment

**Fig. 2:**
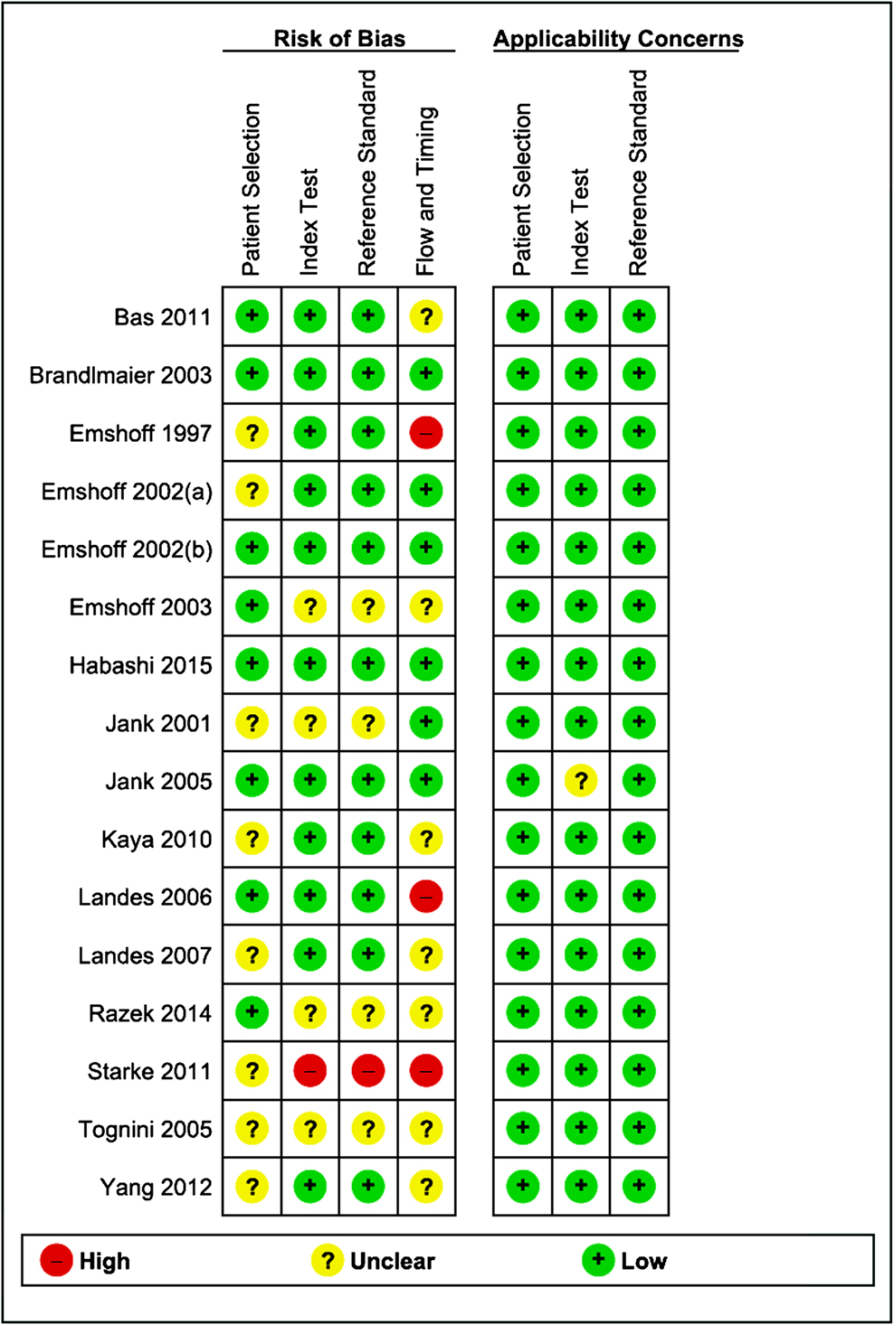
Summary of risk of bias assessment based on QUADAS 2

With respect to the patient selection domain, we consider the risk of bias unclear in eight studies due to poor reporting on sampling procedures [24, 26–28, 30, 32, 34, 35]. For the index test domain, we consider the risk of bias high in one study [35], because the index test results were interpreted without blinding of the reference standard findings. Also, we consider the risk of bias unclear in another four studies [24, 29, 32, 33], because we were unable to confirm whether the index test results were interpreted in a blinded manner of the reference standard findings. For the reference standard domain, we consider the risk of bias high in one study [35], because the reference standard findings were interpreted without blinding of the index test results. The risk of bias in another four studies was considered unclear [24, 29, 32, 33], because we were unable to confirm whether the reference standard results were interpreted in a blinded manner of the index test findings. In the flow and timing domain, we considered the risk of bias high in three studies [23, 28, 35], because not all the patients included were in the final analysis in these studies. The risk of bias in other seven studies was considered unclear [17, 27, 29, 30, 32–34], because the time interval between the index test and the reference standard was not provided. Overall, there were six studies [17, 19, 22, 25, 26, 31] that carried a low risk of bias in all four domains or an unclear risk of bias in only one of the four domains, and there were three studies [23, 28, 35] that carried a high risk of bias in at least one domain (Fig. 2).

The evaluation of the applicability of included studies for the domains of the selection of patients, the index test and the reference standard showed that for only one study [26], insufficient information was provided on the index test. Furthermore, there were no concerns on the evaluated domains of the applicability for all the other included studies (Fig. 2).

### Results of meta-analysis

#### Disc displacement at closed-mouth position

**Fig. 3:**
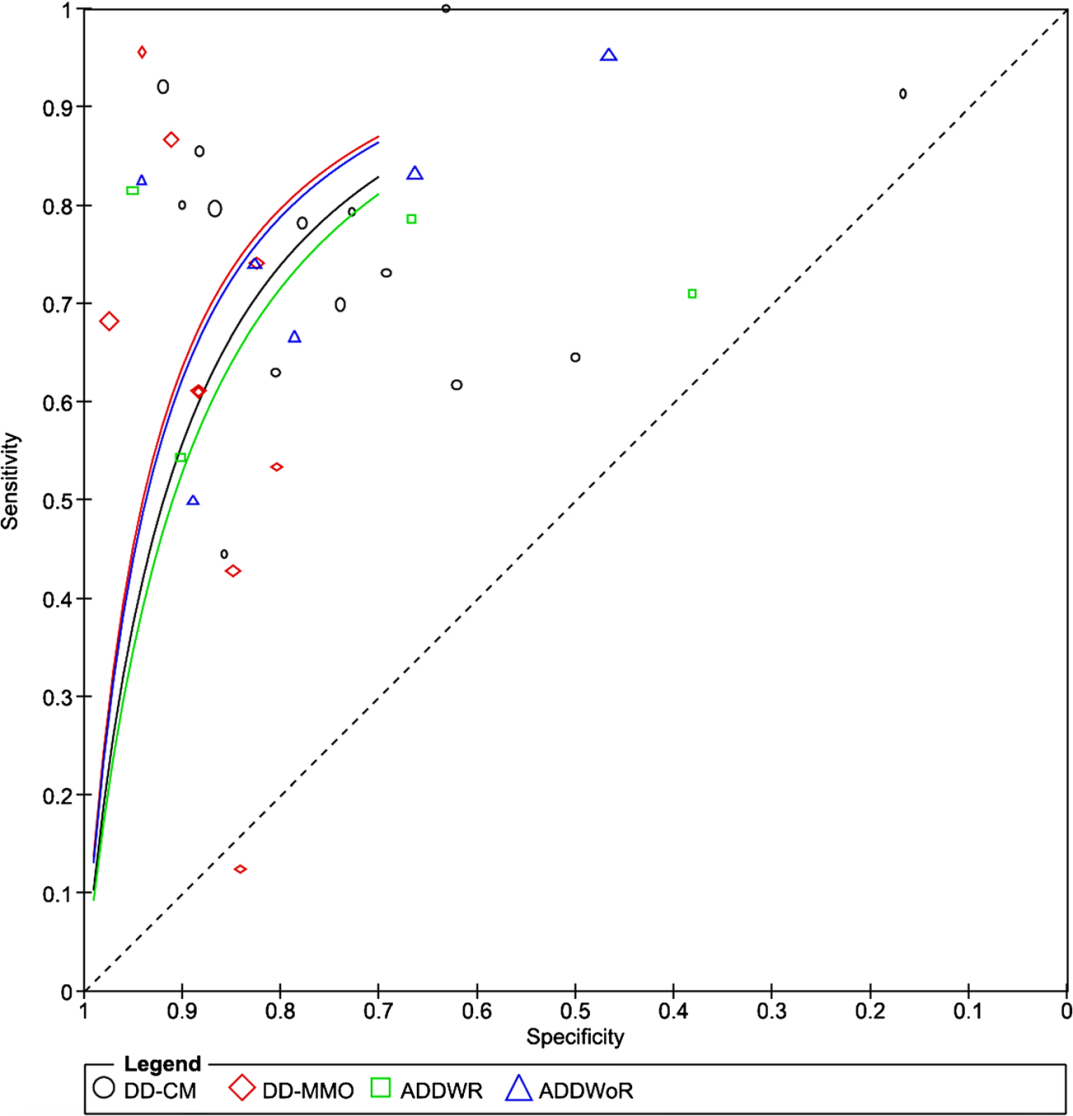
Summary reviewer operator characteristics (sROC) curves. Symbols represent included studies in meta-analyses (*DD-CM* disc displacement at closed mouth position, *DD-MMO* disc displacement at maximum mouth opening, *DDWR* disc displacement with reduction, *DDWoR* disc displacement without reduction)

Fourteen studies were pooled [17, 19, 22–30, 32, 34, 35], and the pooled prevalence (prior probability) of DD-CM was 63% (95%CI: 60–65%). The pooled PPV was 85% (95%CI: 82–87%), and the added value for ruling in an increased risk of DD-CM was 22% (95%CI: 19–26%) (Table 3). The pooled NPV was 68% (95%CI: 64–71%), and the added value for ruling out an increased risk of DD-CM was 30% (95%CI: 26%; 35%) (Table 3). The pooled sensitivity was 0.78 (95%CI: 0.75–0.81), and the pooled specificity was 0.77 (95%CI: 0.73–0.80) (Table 4). Besides, the pooled LR+ was 3.02 (95%CI: 1.96–4.64), and the pooled LR− was 0.32 (95%CI: 0.23–0.45) (Table 4). The summary DOR was 10.80 (95%CI: 5.46–21.38), and the sAUC was 0.836 with a Q* of 0.768 (Fig. 3 and Table 4). When the three studies with high risk of bias [23, 28, 35] were excluded, the pooled added values for ruling in and ruling out an increased risk of DD-CM of the remaining 11 studies were 23% (95%CI: 19–26%) and 33% (28–37%), respectively. The pooled sensitivity was 0.80 (95%CI: 0.77–0.82), and the pooled specificity was 0.79 (95%CI: 0.75–0.82). Besides, the pooled LR+ was 3.31 (95%CI: 1.93–5.66), and the pooled LR− was 0.29 (95%CI: 0.21–0.40). The summary DOR was 12.25 (95%CI: 5.89–25.45), and the sAUC was 0.851 with a Q* of 0.782. The outcomes without the three studies of high risk of bias were very similar to the outcomes with all the studies included.

**Table 3:** Predictive values of ultrasonography for the diagnosis of disc displacement based on MRI

Outcomes	Prevalence		Risk of the presence of DD if the results of US are positive (PPV)			1-Prevalence		Risk of the absence of DD if the results of US are negative (NPV)		
	*n*/*N*	%(95%CI:)	*n*/*N*	%(95%CI:)	Δ% (95%CI:)	*n*/*N*	%(95%CI:)	*n*/*N*	%(95%CI:)	Δ% (95%CI:)
DD-CM (Overall)	922/1469	62.8 (60.3–65.2)	721/849	84.9 (82.4–87.2)	+ 22.2 (18.7–25.6)	547/1469	37.2 (34.8–39.7)	419/620	67.6 (63.8–71.2)	+ 30.4 (25.9–34.8)
DD-CM (Static)	532/853	62.4 (59.1–65.6)	405.5/481.5	84.2 (80.8–87.3)	+ 21.8 (17.3–26.5)	321/853	37.6 (34.4–40.9)	245/371.5	65.9 (60.9–70.5)	+ 28.3 (22.5–34.1)
DD-CM (Static and dynamic)	390/616	63.3 (59.4–67.1)	317/370	85.7 (81.8–89.9)	+ 22.4 (17.2–27.6)	226/616	36.7 (32.9–40.6)	173/246	70.3 (64.4–75.8)	+ 33.6 (26.8–40.5)
DD-CM (≥ 12 MHz)	429/649	66.1 (62.4–69.7)	344/378	91.0 (87.8–93.6)	+ 24.9 (20.3–29.6)	220/649	33.9 (30.3–37.6)	186/271	68.6 (62.9–73.9)	+ 34.7 (28.1–41.4)
DD-CM (< 12 MHz)	208/267	77.9 (72.6–82.6)	148.5/166.5	89.2 (83.7–93.2)	+ 11.3 (4.5–18.2)	59/267	22.1 (17.4–27.4)	41/100.5	40.8 (31.7–50.8)	+ 18.5 (7.7–29.3)
DD-CM (University of Innsbruck)	534/802	66.6 (63.3–69.8)	435.5/471.5	92.4 (89.7–94.5)	+ 25.8 (21.7–29.8)	268/802	33.4 (30.2–36.7)	232/330.5	70.2 (65.2–75.0)	+ 36.7 (30.8–42.6)
DD-CM (Other centers)	388/667	58.2 (54.4–61.9)	285.5/377.5	75.6 (71.1–79.8)	+ 17.5 (11.8–23.2)	279/667	41.8 (38.1–45.6)	187/289.5	64.6 (58.8–69.8)	+ 22.7 (16.0–29.3)
DD-MMO (Overall)	384/1087	35.3 (32.5–38.2)	267/348	76.7 (72.1–80.9)	+ 41.4 (36.1–46.7)	703/1087	64.7 (61.8–67.5)	622/739	84.2 (81.4–86.7)	+ 19.5 (15.6–23.4)
DD-MMO (Static)	290/809	35.9 (32.6–39.2)	190/255	74.5 (68.9–79.6)	+ 38.7 (32.4–45.0)	519/809	64.1 (60.8–67.4)	454/554	81.9 (78.6–85.0)	+ 17.8 (13.2–22.4)
DD-MMO (Static and dynamic)	94/278	33.8 (28.4–39.5)	77/95	81.1 (72.2–88.0)	+ 47.2 (37.6–56.9)	184/278	66.2 (60.5–71.6)	166/183	90.7 (85.8–94.3)	+ 24.5 (17.6–31.5)
DD-MMO (≥ 12 MHz)	246/609	40.4 (36.5–44.3)	172/209	82.3 (76.7–87.0)	+ 41.9 (35.4–48.4)	363/609	59.6 (55.7–63.4)	326/400	81.5 (77.5–85.1)	+ 21.9 (16.5–27.3)
DD-MMO (< 12 MHz)	8/27	29.6 (14.8–48.6)	1/4	25.0 (1.3–75.8)	− 4.6 (−50.4–41.2)	19/27	70.4 (51.4–85.2)	16/23	70.0 (48.9–85.6)	− 0.8 (−26.3–24.7)
DD-MMO (University of Innsbruck)	307/796	38.6 (35.2–42.0)	217/267	81.3 (76.3–85.6)	+ 42.7 (36.9–48.5)	489/796	61.4 (58.0–64.8)	439/529	83.0 (79.6–86.0)	+ 21.6 (16.9–26.2)
DD-MMO (Other centers)	77/291	26.5 (21.6–31.8)	50/81	61.7 (50.8–71.8)	+ 35.3 (23.5–47.0)	214/291	73.5 (68.2–78.4)	183/210	87.1 (82.1–91.2)	+ 13.6 (6.8–20.4)
DDWR	135/354	38.1 (33.2–43.3)	96/132	72.7 (64.7–79.8)	+ 34.6 (25.5–43.7)	219/354	61.9 (56.7–66.8)	183/222	82.4 (77.0–87.0)	+ 20.6 (13.5–27.7)
DDWoR	205/490	41.8 (37.5–46.2)	162/196	82.7 (76.9–87.5)	+ 40.8 (34.0–47.7)	285/490	58.2 (53.8–62.5)	251/294	85.4 (81.0–89.1)	+ 27.2 (21.3–33.2)

**Table 4:** Pooled statistics of ultrasonography for the diagnosis of disc displacement based on MRI using meta-analyses

Outcomes	Number of studies	Sensitivity	Specificity	LR+	LR−	DOR	AUC (SE)	Q* (SE)
DD-CM (Overall)	14	0.78 (0.75–0.81)	0.77 (0.73–0.80)	3.02 (1.96–4.64)	0.32 (0.23–0.45)	10.80 (5.46–21.38)	0.8359 (0.0348)	0.7681 (0.0321)
Static	8	0.76 (0.72–0.80)	0.76 (0.71–0.81)	3.04 (1.69–5.47)	0.35 (0.24–0.52)	9.08 (3.64–22.61)	0.8016 (0.0494)	0.7373 (0.0432)
Static and dynamic	6	0.81 (0.77–0.85)	0.77 (0.70–0.82)	2.84 (1.42–5.66)	0.27 (0.14–0.55)	12.48 (3.79–41.06)	0.8551 (0.0653)	0.7861 (0.0624)
≥ 12 MHz	5	0.80 (0.76–0.84)	0.85 (0.79–0.89)	4.85 (3.49–6.73)	0.24 (0.19–0.29)	21.41 (13.69–33.48)	0.8670 (0.0254)	0.7975 (0.0249)
< 12 MHz	3	0.71 (0.65–0.77)	0.69 (0.56–0.81)	1.88 (0.69–5.12)	0.49 (0.34–0.71)	5.80 (2.93–11.50)	0.7652 (0.0432)	0.7061 (0.0362)
University of Innsbruck	5	0.82 (0.78–0.85)	0.87 (0.82–0.90)	5.75 (3.74–8.85)	0.23 (0.13–0.42)	28.27 (11.68–68.42)	0.9311 (0.0276)	0.8664 (0.0333)
Other centers	9	0.74 (0.69–0.78)	0.67 (0.61–0.73)	2.04 (1.47–2.84)	0.43 (0.33–0.56)	5.30 (3.05–9.23)	0.7566 (0.0355)	0.6989 (0.0296)
DD-MMO (Overall)	9	0.70 (0.65–0.74)	0.88 (0.86–0.91)	5.08 (3.36–7.68)	0.40 (0.26–0.63)	13.53 (6.27–29.18)	0.9523 (0.0213)	0.8936 (0.0292)
Static	7	0.66 (0.60–0.71)	0.87 (0.84–0.90)	4.27 (2.52–7.24)	0.48 (0.30–0.76)	9.72 (3.92–24.06)	0.9546 (0.0309)	0.8968 (0.0431)
Static and dynamic	2	0.82 (0.73–0.89)	0.90 (0.85–0.94)	7.58 (4.07–14.13)	0.25 (0.08–0.79)	29.91 (5.44–164.3)	________	________
≥ 12 MHz	4	0.70 (0.64–0.76)	0.90 (0.86–0.93)	6.36 (3.93–10.28)	0.35 (0.24–0.49)	20.69 (9.61–44.53)	0.9415 (0.0429)	0.8794 (0.0548)
< 12 MHz	1	________	________	________	________	________	________	________
University of Innsbruck	5	0.71 (0.65–0.76)	0.90 (0.87–0.92)	6.12 (3.77–9.94)	0.38 (0.20–0.73)	17.14 (6.98–42.09)	0.9498 (0.0275)	0.8903 (0.0371)
Other centers	4	0.65 (0.53–0.75)	0.86 (0.80–0.90)	3.85 (2.15–6.90)	0.47 (0.26–0.84)	10.30 (2.95–35.97)	0.9638 (0.0315)	0.9102 (0.0477)
DDWR	4	0.71 (0.63–0.79)	0.84 (0.78–0.88)	3.76 (1.26–11.18)	0.40 (0.24–0.68)	9.79 (2.24–42.87)	0.7946 (0.0770)	0.7312 (0.0668)
DDWoR	6	0.79 (0.73–0.84)	0.88 (0.84–0.92)	6.39 (3.46–11.78)	0.26 (0.15–0.46)	29.18 (9.35–91.07)	0.9375 (0.0379)	0.8743 (0.0474)

**Fig. 4:**
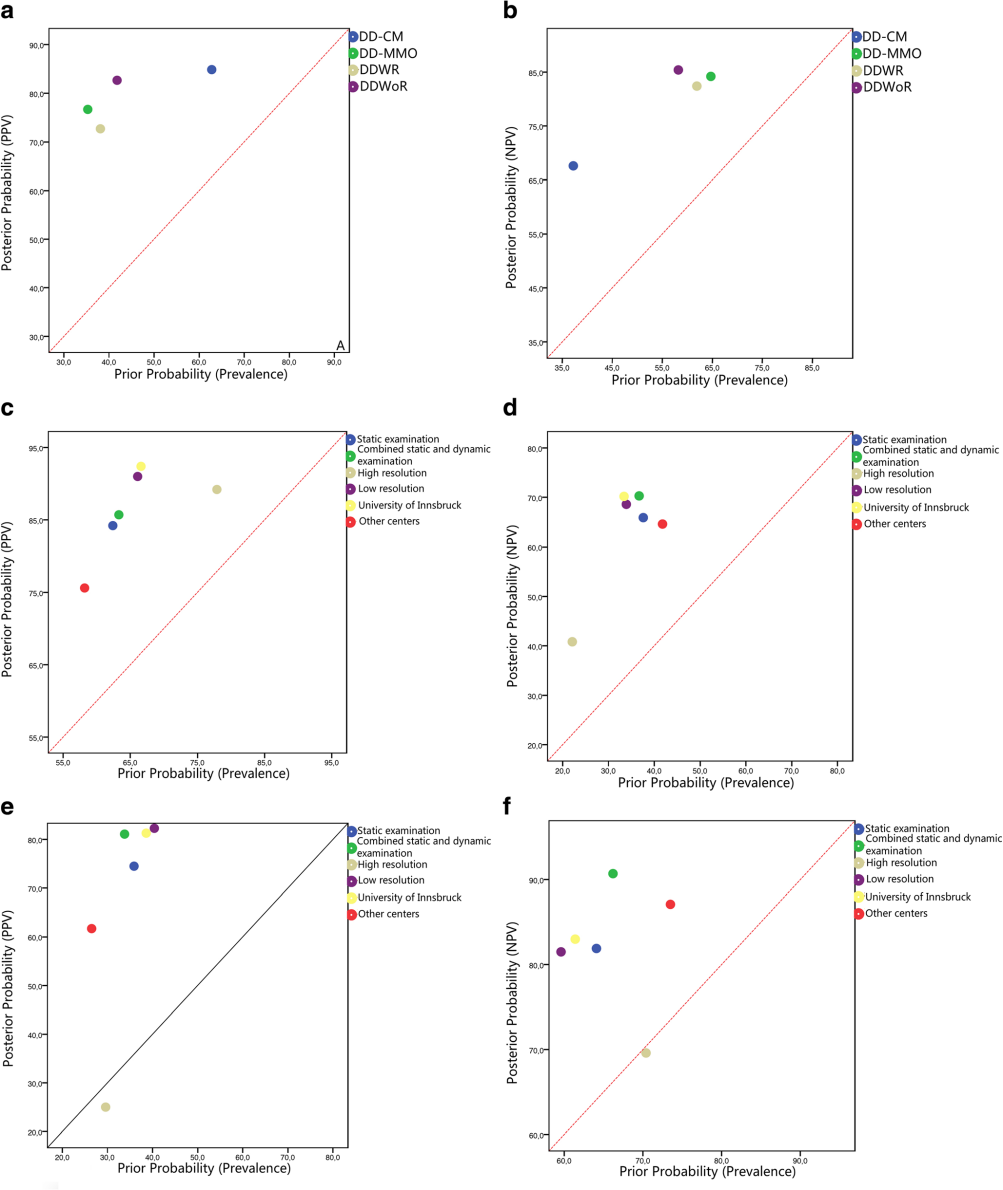
Pooled posterior probabilities of ultrasonography for ruling in or ruling out disc displacement compared to prevalences of disc displacement (**a** positive predictive values of ultrasonography for ruling in the presence of disc displacement at closed mouth position (DD-CM), disc displacement at maximum mouth opening (DD-MMO), disc displacement with reduction (DDWR), and disc displacement without reduction (DDWoR); **b** negative predictive values of ultrasonography for ruling out the presence of DD-CM, DD-MMO, DDWR, and DDWoR; **c** positive predictive values of ultrasonography for ruling in the presence of DD-CM based on subgroups; **d** negative predictive values of ultrasonography for ruling out the presence of DD-CM based on subgroups; **e** positive predictive values of ultrasonography for ruling in the presence of DD-MMO based on subgroups; **f** negative predictive values of ultrasonography for ruling out the presence of DD-MMO based on subgroups). The diagonals represent the posterior probability (PPV or NPV) were the same as the prior probability (prevalence), and there was no added values for ruling in or ruling out the DDs at this diagonal

The subgroup analyses were performed for the types of US (static and/or dynamic), the resolution of US (high resolution or low resolution) and the centers of the researches (University of Innsbruck or other centers). The pooled added values for ruling in and out an increased risk of DD-CM based on the subgroups are presented in Table 3 and Fig. 4. The pooled sensitivity, specificity, LR+, LR−, DOR, and sAUC are presented in Table 4.

#### Disc displacement at maximum mouth-opening position

Nine studies were pooled [19, 22–28, 30], and the pooled prevalence (prior probability) of DD-MMO was 35% (95%CI: 33–38%). The pooled PPV was 77% (95%CI: 72–81%), and the added value for ruling in an increased risk of DD-MMO was 41% (95%CI: 36–47%) (Table 3). The pooled NPV was 84% (95%CI: 81–87%), and the added value for ruling out an increased risk of DD-MMO was 20% (95%CI: 16–23%) (Table 3). The pooled sensitivity was 0.70 (95%CI: 0.65–0.74), and the pooled specificity was 0.88 (95%CI: 0.86–0.91) (Table 4). Besides, the pooled LR+ was 5.08 (95%CI: 3.36–7.68), and the pooled LR− was 0.40 (95%CI: 0.26–0.63) (Table 4). The summary DOR was 13.53 (95%CI: 6.27–29.18), and the sAUC was 0.952 with a Q* of 0.894 (Fig. 3 and Table 4). When the two studies [23, 28] with high risk of bias were excluded, the pooled added values for ruling in and ruling out an increased risk of DD-CM of the remaining seven studies were 43% (95%CI: 37–48%) and 22% (18–26%), respectively. The pooled sensitivity of the remaining seven studies was 0.72 (95%CI: 0.67–0.77), and the pooled specificity was 0.89 (95%CI: 0.86–0.92). Besides, the pooled LR+ was 5.95 (95%CI: 3.99–8.85), and the pooled LR− was 0.33 (95%CI: 0.23–0.47). The summary DOR was 19.74 (95%CI: 9.60–40.62), and the sAUC was 0.947 with a Q* of 0.886. The outcomes without the two studies of high risk of bias were very similar to the outcomes with all the studies included.

The subgroup analyses were performed for the types of US (static and/or dynamic), the resolution of US (high resolution or low resolution) and the centers of the researches (University of Innsbruck or other centers). The pooled added values for ruling in and out an increased risk of DD-MMO based on the subgroups are presented in Table 3 and Fig. 4. The pooled sensitivity, specificity, LR+, LR−, DOR, and sAUC are presented in Table 4.

#### DDWR

Four studies were pooled [19, 25, 31, 34], and the pooled prevalence (prior probability) of DDWR was 38% (95%CI: 33–43%). The pooled PPV was 73% (95%CI: 65–80%) and the added value for ruling in an increased risk of DDWR was 35% (95%CI: 26–44%) (Table 3). The pooled NPV was 82.4% (95%CI: 77–87%), and the added value for ruling out an increased risk of DDWR was 21% (95%CI: 14–28%) (Table 3). The pooled sensitivity was 0.71 (95%CI: 0.63–0.79), and the pooled specificity was 0.84 (95%CI: 0.78–0.88) (Table 4). Besides, the pooled LR+ was 3.76 (95%CI: 1.26–11.18), and the pooled LR− was 0.40 (95%CI: 0.24–0.68) (Table 4). The summary DOR was 9.79 (95%CI: 2.24–42.87), and the sAUC was 0.7946 with a Q* of 0.731 (Fig. 3 and Table 4).

#### DDWoR

Six studies were pooled [19, 25, 27, 31, 33, 34], and the pooled prevalence (prior probability) of DDWoR was 42% (95%CI: 38–46%). The pooled PPV was 83% (95%CI: 77–88%), and the added value for ruling in an increased risk of DDWoR was 41% (95%CI: 34–48%) (Table 3). The pooled NPV was 85% (95%CI: 81–89%), and the added value for ruling out an increased risk of DDWoR was 27% (95%CI: 21–33%) (Table 3). The pooled sensitivity was 0.79 (95%CI: 0.73–0.84) and the pooled specificity was 0.88 (95%CI: 0.84–0.92) (Table 4). Besides, the pooled LR+ was 6.39 (95%CI: 3.46–11.78), and the pooled LR− was 0.26 (95%CI: 0.15–0.46) (Table 4). The summary DOR was 29.18 (95%CI: 9.35–91.07), and the sAUC was 0.938 with a Q* of 0.874 (Fig. 3 and Table 4).

## Discussion

The findings in this systematic review indicate that US has sufficient added value for both ruling in and ruling out DD. With the use of US, the probabilities for ruling in or ruling out DDs may increase, with a difference of about 20% or more between prior and posterior probability.

Radiographic imaging can be used for confirming the diagnosis of DD after clinical examination. That is, the ability of US for ruling out a DD is important in selective cases in dental practice based on the Diagnostic Criteria for Temporomandibular Disorders (DC/TMD) [10]. In the present systematic review, if a patient had suspected DDs symptoms and signs or had a clinical diagnosis of DDs, the prior probability of not having DD at closed-mouth position and maximum mouth-opening position is 37.2 and 64.7%, respectively. With a negative US result, the posterior probability of not having a DD is increased to 67.6 and 84.2%. Hence, the negative results of US have good added values for ruling out a DD and may help dentists to confirm the absence of a DD when indicated.

For dentists and patients, it is important to confirm the diagnosis of DD after clinical examinations in selective cases, for example, in patients who experience a substantial influence of the DDs on their oral health-related quality of life (OHRQoL). For policy makers, such information could provide evidence whether US should be used in dental practice as a screening tool after clinical examinations to confirm the diagnosis of DD. For researchers, US can replace MRI for the diagnosis of DDs because US is cheaper and more accessible.

Besides, in the present systematic review, the sROCs of DD-CM, DD-MMO, DDWR, and DDWoR ranged from 0.79 to 0.95, which indicated that the diagnostic values of US for the diagnosis of DD are good to excellent [22, 36]. Also, sensitivity and specificity of US for the diagnosis of DD were presented in the review. The sensitivity of US for the diagnosis of DD ranged from 0.70 to 0.79, while the specificity ranged from 0.77 to 0.88. Except for DD-CM, the specificity was significantly higher than the sensitivity, which also suggests that the US has better ability to exclude those patients without actual DD than to include those with actual DD, and this result is consistent with a previous review [15]. However, for clinical decision-making, the sensitivity and specificity are not very helpful, because they only indicate how good a test discerns cases from non-cases.

**Fig. 5:**
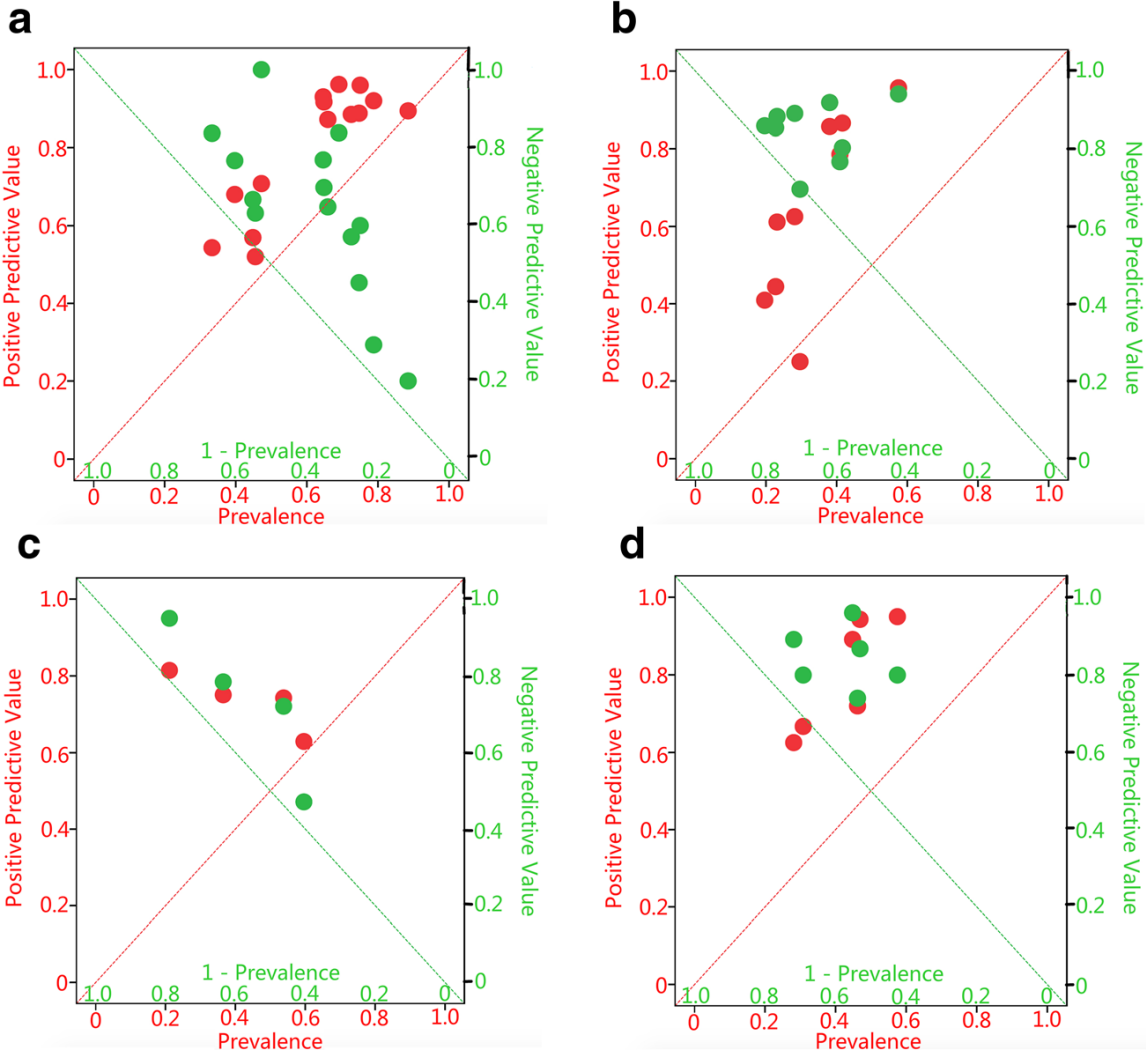
Positive and negative predictive values of ultrasonography compared to the prevalences of disc displacement at closed mouth position (DD-CM), disc displacement at maximum mouth opening (DD-MMO), disc displacement with reduction (DDWR), and disc displacement without reduction (DDWoR) for each included study. Red circles represent positive predictive values of US of the included studies and green circles represent negative predictive values of US of the included studies. (**a** DD-CM, **b** DD-MMO, **c** DDWR, **d** DDWoR). The red diagonals represent the positive predictive value were the same as prevalence, and there was no added value for ruling in the DDs at this diagonal. The green diagonals represent the negative predictive value were the same as the compliment of prevalence (1—prevalence), and there was no added value for ruling out the DDs at this diagonal

The present review shows that for almost all the included studies, the PPVs and NPVs for DD-CM, DD-MMO, DDWR, and DDWoR exceeded the prior probabilities to a large extent (see Fig. 5). This indicates that the added values of US for DD are sufficient. However, there was an exception that the PPV and NPV were both below the diagonal in one study in DD-MMO. This means that, compared to MRI, US resulted in major misclassification and so in a large number of false-positive and false-negative findings. This suggests that something might have gone wrong in that particular study [28]. A possible explanation might be that this was the first study (published in 1997) exploring the use of US in the diagnosis of DD. So, the researchers may still have been rather inexperienced and experimenting with the use of US and exploring its interpretation. Moreover, in this particular study, low-resolution US was used, which may have contributed to the low accuracy of US [15, 22, 36].

In this systematic review, we evaluated the accuracy of US for DD-CM and DD-MMO in several subsamples of included studies. Dynamic examination is regarded as “real-time,” as it allows dentists to detect the disc position in more detail during mouth opening and jaw movement [22, 36]. However, there are no studies published on the diagnostic value of dynamic examination alone. Still, the added diagnostic values of the combined static and dynamic examinations of US for ruling in and ruling out DD-CM and DD-MMO tended to be higher than for static examination alone. High-resolution US allows better visualization of joint structures than low-resolution US [15]. Current evidence shows that the added diagnostic values of US for ruling in and ruling out DD are both higher than with low-resolution US.

To date, a protocol for using and interpreting US in DD has neither been established nor has a recommendation for this been published. In the 16 included studies, six studies adopted vertical scans, three adopted horizontal scans, five adopted combined vertical and horizon scans, and one chose 60 degrees to the Frankfort plane as the initial position to scan. In the included studies, the definitions of the disc in the US images are not the same and ranged from hyperechogenic and isoechogenic to hypoechogenic. Hence, there seems to be neither a consensus on a standardized position of US in TMJ areas, nor a prevailing standardized approach for its interpretation.

The performance of US in clinical practice is reported to be operator-dependent [16]. Visualization of the disc through US can be technically challenging, because the disc can be visualized only through the small gap between zygomatic process of the temporal bone and the top of the condyle [16]. However, constantly adjusting the position of the transducers and well-trained operators are thought to be helpful to obtain better visualization of the disc [16]. So, training and calibration are necessary in operating US and interpreting images in clinical practice [16]. We found that seven of the included 16 studies have been reported by the University of Innsbruck. It has been the first center to study the diagnostic utility of US for DD [28], and the investigators in this center clearly were focused on this. So they may have gained more experience in using US and interpreting its findings. Interestingly, the added diagnostic value of US in these seven studies from the University of Innsbruck tended to be higher than those performed in the other centers.

In interpreting the findings of the present systematic review, there are some limitations that should be taken in consideration.

First, MRI was used as the reference standard in the review. However, MRI may not have perfect diagnostic accuracy for diagnosis of DD. Compared to cryosectional morphology of the TMJ on autopsy, the sensitivity of MRI ranged from 0.75 to 0.90, while the specificity ranged from 0.80 to 1.00 for the diagnosis of TMJ disc position [37–39]. Unfortunately, no comparison has been made with US in such autopsy studies. Still, MRI can be regarded as the reference standard for diagnosis of DDs for pragmatic reasons.

Second, while publication bias cannot be excluded, we did not search for unpublished research. Moreover, there is no registry or mechanism of registration for designs of diagnostic accuracy studies such as with randomized trials. As such, it will be difficult to retrieve unpublished diagnostic accuracy studies.

Third, in the 16 included studies of the present systematic review, seven studies were from the same institution (University of Innsbruck) from 1997 to 2005. However, five of them did not report the starting and end date of the researches so we cannot confirm whether the same subjects were participated in more than one study in these five studies. If that was the case, it may affect the results of meta-analyses and cause potential bias.

It should be mentioned that there are two systematic reviews published in 2012 and 2015 on the same topic as the present systematic review [13, 40]. These two previous systematic reviews both showed that US can be accepted as a rapid preliminary diagnostic method for DD, which is similar as the results of the present systematic review. However, these two previous studies mainly focused on the sensitivity and specificity of US based on MRI, which is meaningful for researchers but cannot be directly used in clinical practice for dentists. In the present study, we showed US is an acceptable method for diagnosis of DD in clinical practice based on the added values of PPV and NPV of US. That is, the first-line dentists can make decisions about the risk of an individual having a diagnosis of DD directly based on our results. Besides, for the previous systematic reviews, both follow-up studies and case-control studies were included for meta-analyses. However, in the present systematic review, we only included follow-up studies because case-control studies cannot truly reflect the prevalence, PPV, NPV, sensitivity, and specificity, and can be prone to bias [41] so that we cannot obtain the real added values of US for ruling in and ruling out DD. So, the results of the present systematic review should be more reliable than the previous ones.

For the future studies and clinical practice, a standardized protocol for the diagnostic use of US in patients suspected of DD should be established, and US operators should be well-trained and calibrated to reduce the variations in US across operators and studies. Also, additional higher quality studies are needed for further assessment of the value of different types of US for diagnosis of DD. Besides, in the future studies, improvement of the reference standard that with higher certainty results in an accurate external benchmark for the presence of TMJ DD clinical practice should be considered. If this is not available, a latent variable approach can be adopted for data analysis, by combining multiple diagnostic tests using a latent class modeling (LCM) [42]. This may help to improve the reliability of US for ruling in and out TMJ DD.

## Conclusions

With MRI being used as the reference standard, the added diagnostic value of US for ruling in and ruling out DD is sufficient for its use in the decision-making in dental practice.

After sufficient training and calibration of operating and interpretation, US can be considered as a relevant imaging tool to supplement clinical examination in patients with suspected DDs in selected cases. Combined static and dynamic examinations using high-resolution US should be preferred.
